# Pan‐Immune‐Inflammation Value: Related to Perforation Diameter and Pulmonary Artery Pressure in Ventricular Septal Rupture Patients

**DOI:** 10.1155/mi/5407966

**Published:** 2026-04-08

**Authors:** Xinlong Di, Zebin Lin, Qingwang Hou, Tongfeng Chen, Xiaohu Wang, Chong Chen, Jianmin Tang, Yuhao Liu, Yipin Zhao

**Affiliations:** ^1^ Department of Cardiology, The Second Affiliated Hospital of Zhengzhou University, Zhengzhou, 450014, China, zzusah.com; ^2^ Department of Geriatrics, Zhongshan Hospital Xiamen University, 201 Hubin South Road, Xiamen, Fujian, China, xmu.edu.cn; ^3^ Department of Cardiology, Henan University People’s Hospital, Henan Provincial People’s Hospital, Zhengzhou, 450000, China, hnsrmyy.net; ^4^ Department of Cardiology, Fuwai Central China Cardiovascular Hospital, Central China Fuwai Hospital of Zhengzhou University, Zhengzhou, 451464, Henan, China

**Keywords:** acute myocardial infarction, pan-immune-inflammation value, perforation diameter, pulmonary hypertension, ventricular septal rupture

## Abstract

**Objective:**

This study investigated the relationship between the pan‐immune‐inflammation value (PIV) and the perforation diameter in patients with ventricular septal rupture (VSR), as well as the changes in pulmonary artery systolic pressure (*Δ*PASP) after transcatheter closure.

**Methods:**

The clinical data from 133 VSR patients who underwent transcatheter closure were analyzed. Patients were divided into high and low PIV groups based on a median cutoff value of 6. Boruta was used for exploratory variable screening; prespecified covariate sets were used for adjusted models. A generalized additive model (GAM) was used to explore the potential nonlinear relationships. Where a nonlinear association was suggested by the GAM, a piecewise linear regression model was subsequently fitted to precisely quantify the threshold effects and the differential associations of PIV/100 with *Δ*PASP and perforation diameter on either side of the identified inflection point.

**Results:**

One hundred thirty‐three patients with VSR (mean age 67 years, 50.6% female) were enrolled. Linear regression showed no statistically significant association between PIV/100 and *Δ*PASP. GAM suggested a possible nonlinear pattern between PIV/100 and *Δ*PASP (and perforation diameter). In piecewise models, an inverse association was observed above the break point, but this pattern was attenuated after multivariable adjustment and in sensitivity analyses. In discrimination analyses, adding PIV/100 to the baseline model yielded a modest numerical increase in AUC, but the incremental gain was not statistically significant.

**Conclusion:**

PIV may serve as an adjunct inflammatory marker in VSR patients undergoing transcatheter closure; however, the break point findings are exploratory and the incremental predictive value warrants validation in larger, independent cohorts with better adjudication of infection/systemic inflammation.

## 1. Introduction

Ventricular septal rupture (VSR) is still a fatal mechanical complication of acute myocardial infarction (AMI) [1]. Although the prevalence rate has significantly decreased, we have had little impact on the natural history and severe prognosis associated with the disease. In the era before thrombolytic therapy, more than 2% of all cases of AMI exhibited VSR. The introduction of early thrombolysis and percutaneous coronary revascularization significantly reduced the incidence of VSR, far below 1% [2]. Mortality with this complication remains extremely high in the thrombolytic era, despite improvements in medical therapy and percutaneous and surgical techniques [3]. Patients with VSR frequently exhibit elevated pulmonary artery pressure. However, following transcatheter occlusion, a significant reduction in pulmonary artery pressure is observed, thereby minimizing the proportion of patients requiring pharmacological treatment for pulmonary hypertension.

Inflammation is a prominent feature of human and experimental pulmonary arterial hypertension (PH), manifesting as infiltration of various inflammatory cells and increased expression of specific cytokines during the remodeling of pulmonary vessels [4]. Neutrophils play an important role in pulmonary arterial hypertension as well [5]. The occurrence of many cardiovascular events also involves inflammatory factors [6]. Studies have suggested that the pathological characteristics of myocardial infarction provide an understanding of the cellular signaling pathways involved in the survival, growth, apoptosis, and autophagy of cardiomyocytes, endothelial cells, fibroblasts, monocytes, and stem cells. These pathways encompass key elements of the inflammatory response [7–11]. The pan‐immune‐inflammation value (PIV) serves as a marker of systemic inflammation. Compared to other immune indicators like the neutrophil to lymphocyte ratio (NLR), monocyte to lymphocyte ratio (MLR), and platelet (PLT) to lymphocyte ratio (PLR), PIV may provide a more comprehensive assessment of inflammation. This is attributed to PIV integrating the counts of four major immune cell types in peripheral blood: neutrophils, monocytes, PLTs, and lymphocytes [12–14]. In addition, cardiometabolic disorders are closely interrelated and may contribute to shared inflammatory, vascular, and end‐organ injury pathways in cardiovascular disease [15].

Although some inflammatory blood‐cell indices have been reported to predict cardiac rupture after AMI, evidence regarding PIV in VSR patients after transcatheter closure remains limited [16]. This study examines the correlation between PIV, perforation diameter, and postoperative pulmonary hypertension in patients with VSR, intending to identify a reliable parameter for assessing perforation size and predicting the occurrence of postoperative pulmonary hypertension. Therefore, this study was designed to move beyond simple linear associations. We hypothesized that the relationships between systemic inflammation (as measured by PIV), anatomical defect size, and postoperative hemodynamic changes are complex and nonlinear. To test this, we employed generalized additive models (GAMs) to rigorously explore and characterize these potential nonlinear dynamics.

## 2. Materials and Methods

### 2.1. Study Population

This study is a retrospective cross‐sectional study conducted at Central China Fuwai Hospital of Zhengzhou University. Patients with postinfarction VSR (PIVSR) admitted to the hospital between January 2018 and September 2024 were enrolled. All blood samples for PIV calculation were collected within 24 h of VSR diagnosis, before any interventional procedure, to reflect the acute inflammatory state associated with the rupture event. Among the initially identified 248 PIVSR patients, 31 underwent surgical repair and 62 received conservative treatment or did not meet surgical criteria. Another 22 patients were excluded due to incomplete clinical data. Finally, 133 patients who successfully closed the ruptured interventricular septum through catheterization were included in the analysis. All included patients met the diagnostic criteria for AMI. The echocardiogram indicates a clear interruption of interventricular septal echo continuity and left‐to‐right shunting at the level of perforation.

Given the retrospective design of this study, informed consent has been waived. All procedures involving human participants are conducted in accordance with the Helsinki Declaration (revised in 2013). All procedures are conducted in accordance with applicable guidelines and regulations.

### 2.2. Selection Criteria and Definitions

#### 2.2.1. Inclusion Criteria

(1) Patients diagnosed with AMI combined with ventricular septal perforation according to the 2018 European AMI guidelines, including acute ST‐segment elevation myocardial infarction (STEMI) and acute non‐STEMI. Diagnostic criteria for VSR: (1) Physical examination: Loud and newly emerging systolic murmurs, sometimes accompanied by tremors, can be heard between the our and five intercostal spaces on the left edge of the sternum. (2) Echocardiography revealed interrupted continuity of interventricular septal echoes and left‐to‐right shunting. (3) All patients diagnosed with VSR underwent closure of the perforation of the interventricular septum.

#### 2.2.2. Exclusion Criteria

(1) VSR caused by nonmyocardial infarction factors; (2) due to other reasons, coronary artery bypass grafting, heart valve replacement, or cardiac surgery is required; (3) lack of clinical data; (4) patients with documented active infection/sepsis based on medical records were excluded when available; however, procalcitonin and microbiological culture were not routinely obtained in this retrospective cohort.

### 2.3. Calculation of Evaluation Indicators

As described above, blood samples were obtained at VSR diagnosis prior to intervention. Pulmonary artery systolic pressure (PASP) was measured by transthoracic echocardiography before and after transcatheter closure. The PIV was calculated as:

PIV=Neutrophil count×platelet count×monocyte countLymphocyte count.

All cell counts are expressed in ×10^3^ cells/μL. To improve numerical stability and interpretability of regression coefficients, we used PIV/100 in all analyses, consistent with prior literature employing scaled composite inflammatory indices [17]. Accordingly, the threshold of PIV/100 = 6.36 corresponds to a raw PIV = 636.

The change in PASP (*Δ*PASP) was defined as:

ΔPASP=Preoperative PASP−postoperative PASP.

A positive value indicates a reduction in PASP following transcatheter closure.

### 2.4. Data Collection

Demographic and clinical information were retrieved from the hospital’s electronic medical record system. All laboratory tests and examinations are conducted within 24 h after the diagnosis of VSR. The collected data includes: main clinical variables include: 1. Basic information of patients (such as age, gender, etc.). 2. Medical history: hypertension, diabetes, ventricular aneurysm, smoking, and drinking. 3. Laboratory testing: blood cell count (WBC), PLT count, NT‐proBNP, hs‐CTnI, myoglobin (MYo), neutrophil (N), lymphocyte (L), monocyt (M), glucose (Glu), glycated hemoglobin A1c (HBA1c), albumin (ALB), alanine aminotransferase (ALT), aspartate aminotransferase (AST), lactate dehydrogenase (LDH), direct bilirubin (DBIL), total bilirubin (TBIL), D–dimer(D–D), international normalized ratio (INR), activated partial thromboplastin time (APTT), total cholesterol (TC), low‐density lipoprotein (LDL), triglyceride (TG), blood urea nitrogen (BUN), perforative diameter, PASP, intra‐aortic balloon pump (IABP), continuous renal replacement therapy (CRRT), extracorporeal membrane oxygenation (ECMO), and so on. 4. Echocardiographic results: Perforation of interventricular septum location, perforation diameter, left ventricular ejection fraction (LVEF), PASP, and so on.

### 2.5. Statistical Analysis

For descriptive statistics, normally distributed variables were presented as mean (standard deviation [SD]), nonnormally distributed continuous variables as median (interquartile range [IQR]), and categorical variables as frequency (percentage). Normality tests and *Q*–*Q* plots were used to assess data distribution, with appropriate descriptive statistical methods applied for variables with normal or non‐normal distributions. For intergroup comparisons: normally distributed continuous variables were analyzed using the Welch *t*‐test or analysis of variance (ANOVA); nonnormally distributed continuous variables were compared using the Wilcoxon rank–sum test or Kruskal–Wallis test; for categorical variables, Fisher’s exact test was employed when the expected frequency was <5, otherwise the chi‐square test was used. Candidate predictors were screened using the Boruta algorithm as an exploratory variable‐importance procedure. Boruta was used for exploratory screening; final multivariable models were prespecified for confounder adjustment based on clinical plausibility and parsimony. A Boruta‐augmented sensitivity analysis additionally including TBIL/AST/ALT was performed (Table S9). GAMs were used to explore potential nonlinear associations between PIV/100 and perforation diameter as well as *Δ*PASP. When the GAM suggested a potential nonlinear pattern, segmented regression (R package “segmented”) was used to identify an inflection point, which was then fixed as the break point for subsequent piecewise linear regression; model fit was compared using the log‐likelihood ratio test. Sensitivity analyses were conducted to assess potential confounding by systemic inflammation and the stability of the exploratory break point, including CRP‐Q4 exclusion and additional adjustment for log (CRP + 1) (Tables S4–S8). Statistical significance was set at *p*  < 0.05 (two‐tailed). All analyses were performed in R (version 4.2.2 or later).

## 3. Results

### 3.1. Baseline Characteristics

According to the inclusion and exclusion criteria, 133 patients with VSR (mean age 67 years, 50.6% female) were enrolled, with their baseline characteristics summarized in Table 1. Patients were stratified into low‐level and high‐level groups using a median PIV/100 value of 6 as the cutoff: the low‐level group (PIV/100 <6) and the high‐level group (PIV/100 ≥6).

**Table 1:** Comparison of baseline data characteristics among different PIV value groups.

Characteristic	PIV/100 group		*p*‐Value
	<6, *N* = 65^a^	≥6, *N* = 68^a^	
Age (years)	67 (62, 71)	67 (64, 72)	0.352^b^
Gender, *n* (%)			0.570^c^
Female	37 (56.9%)	42 (61.8%)	—
Male	28 (43.1%)	26 (38.2%)	—
Hypertension, *n* (%)			0.194^c^
No	36 (55.4%)	30 (44.1%)	—
Yes	29 (44.6%)	38 (55.9%)	—
Diabetes, *n* (%)			0.356^c^
No	46 (70.8%)	43 (63.2%)	—
Yes	19 (29.2%)	25 (36.8%)	—
Ventricular aneurysm, *n* (%)			0.869^c^
No	19 (29.2%)	19 (27.9%)	—
Yes	46 (70.8%)	49 (72.1%)	—
Smoking, *n* (%)			0.448^c^
No	48 (73.8%)	54 (79.4%)	—
Yes	17 (26.2%)	14 (20.6%)	—
Drinking, *n* (%)			0.707^c^
No	56 (86.2%)	57 (83.8%)	—
Yes	9 (13.8%)	11 (16.2%)	—
NT‐proBNP (pg/mL)	5627 (2664, 9546)	8326 (5041, 12,117)	0.014^b^
hs‐CTnI (ng/mL)	126 (20, 416)	671 (205, 1508)	<0.001^b^
Myo (ng/mL)	89 (49, 159)	297 (159, 397)	<0.001^b^
Glucose (mmol/L)	7.8 (5.2, 9.8)	8.2 (6.3, 10.7)	0.076^b^
HBA1c (%)	6.74 (5.87, 7.43)	6.49 (5.89, 7.63)	0.791^b^
WBC (×10^9^/L)	6.8 (5.9, 8.8)	12.2 (9.2, 15.4)	<0.001^b^
RBC (×10^9^/L)	4.00 (3.60, 4.26)	3.90 (3.55, 4.25)	0.451^b^
PLT (×10^9^/L)	187 (153, 239)	255 (189, 299)	<0.001^b^
HB (g/L)	119 ± 18	120 ± 18	0.738^d^
Neutrophil (×10^9^/L)	4.0 (3.2, 5.5)	10.4 (8.6, 13.8)	<0.001^b^
Lymphocyte (×10^9^/L)	1.54 (1.21, 2.38)	1.17 (0.86, 1.62)	<0.001^b^
Monocyte (×10^9^/L)	0.52 (0.41, 0.66)	0.84 (0.70, 1.00)	<0.001^b^
CRP (mg/L)	12 (5, 29)	46 (14, 87)	<0.001^b^
ALB (g/L)	38.0 (35.5, 40.6)	35.7 (33.8, 37.6)	<0.001^b^
ALT (U/L)	25 (15, 55)	43 (22, 129)	0.011^b^
AST (U/L)	25 (18, 57)	63 (29, 280)	<0.001^b^
DBIL (umol/L)	7.8 (5.9, 9.2)	6.6 (4.4, 10.1)	0.088^b^
TBIL (umol/L)	16 (12, 20)	13 (9, 18)	0.029^b^
HCY (umol/L)	24 (17, 32)	20 (16, 29)	0.246^b^
TT (s)	17 (17, 19)	18 (17, 20)	0.129^b^
PT (s)	12.10 (11.30, 13.10)	12.45 (11.60, 14.53)	0.053^b^
D–D (mg/L)	2.6 (1.5, 4.1)	4.0 (1.4, 8.1)	0.026^b^
INR	1.07 (0.97, 1.18)	1.09 (1.00, 1.26)	0.236^b^
APTT (s)	28 (25, 31)	32 (27, 37)	0.003^b^
TC (mmol/L)	3.41 (2.91, 3.86)	3.54 (3.13, 4.39)	0.156^b^
LDL (mmol/L)	2.13 (1.89, 2.45)	2.32 (1.96, 2.65)	0.305^b^
Non‐HDL‐c (mmol/L)	2.58 (2.14, 3.01)	2.72 (2.34, 3.40)	0.071^b^
TG (mmol/L)	1.23 (0.90, 1.60)	1.48 (0.93, 1.80)	0.178^b^
SCR (umol/L)	86 (68, 113)	90 (74, 135)	0.192^b^
BUN (mg/dL)	8 (6, 11)	10 (7, 15)	0.006^b^
UA (umol/L)	419 (327, 488)	386 (334, 575)	0.560^b^
LDH (IU/L)	235 (191, 415)	576 (395, 925)	<0.001^b^
eGFR (mL/min)	60 ± 19	57 ± 19	0.397^d^
Na (mmol/L)	139.0 (136.0, 141.0)	137.0 (134.0, 141.0)	0.082^b^
K (mmol/L)	4.15 ± 0.45	4.29 ± 0.50	0.090^d^
MI to operation time (day)	24 (19, 35)	22 (17, 24)	0.055^b^
Perforative diameter (mm)	13.5 (10.0, 17.0)	14.3 (10.0, 16.1)	0.543^b^
Perforative parts, *n* (%)			0.951^e^
Apex of the ventricular septum	48 (73.8%)	51 (75.0%)	—
Anterior ventricular septum	3 (4.6%)	4 (5.9%)	—
Posterior ventricular septum	14 (21.5%)	13 (19.1%)	—
Target vascular lesions, *n* (%)			0.160^e^
LAD	50 (76.9%)	61 (89.7%)	—
LCX	3 (4.6%)	1 (1.5%)	—
RCA	12 (18.5%)	6 (8.8%)	—
EF (%)	53 (46, 57)	55 (46, 59)	0.452^b^
EF postoperative (%)	53 (48, 59)	54 (47, 58)	0.792^b^
*Δ*EF (%)	1 (−2, 4)	1 (−3, 4)	0.846^b^
PASP (mmHg)	51 ± 17	53 ± 14	0.637^d^
PASP postoperative (mmHg)	43 ± 13	42 ± 13	0.643^d^
*Δ*PASP (mmHg)	8 (−1, 17)	10 (3, 18)	0.259^b^
Deal with the timing, *n* (%)			0.272^c^
Preoperative ventricular septal closure	39 (60.0%)	47 (69.1%)	—
After Ventricular septal closure	26 (40.0%)	21 (30.9%)	—
IABP, *n* (%)			0.007^c^
No	41 (63.1%)	27 (39.7%)	—
Yes	24 (36.9%)	41 (60.3%)	—
CRRT, *n* (%)			0.063^e^
No	64 (98.5%)	61 (89.7%)	—
Yes	1 (1.5%)	7 (10.3%)	—
ECMO, *n* (%)			>0.999^e^
No	62 (95.4%)	64 (94.1%)	—
Yes	3 (4.6%)	4 (5.9%)	—
Transfusion, *n* (%)			0.407^c^
No	50 (76.9%)	48 (70.6%)	—
Yes	15 (23.1%)	20 (29.4%)	—

*Note:* HBA1c, glycated hemoglobin A1c.

Abbreviations: ALB, albumin; ALT, alanine aminotransferase; APTT, activated partial thromboplastin time; AST, aspartate aminotransferase; BUN, blood urea nitrogen; CRP, C‐reactive protein; CRRT, continuous renal replacement therapy; DBIL, direct bilirubin; D–D, D‐dimer; ECMO, extracorporeal membrane oxygenation; EF, ejection fraction; eGFR, estimated glomerular filtration rate; Glu, glucose; HB, hemoglobin; HCY, homocysteine; hs‐CTnl, high‐sensitivity cardiac troponin I; IABP, intra‐aortic balloon pump; INR, international normalized ratio; L, lymphocyte; LDH, lactate dehydrogenase; LDL, low‐density lipoprotein; M, monocyte; MI, myocardial infarction; Myo, myoglobin; N, neutrophil; non‐HDL‐c, non‐high‐density lipoprotein cholesterol; NT‐proBNP, N‐terminal prohormone of brain natriuretic peptide; PASP, pulmonary artery systolic pressure; PLT, platelet; PT, prothrombin time; RBC, red blood cell; SCR, serum creatinine; TBIL, total bilirubin; TC, total cholesterol; TG, triglyceride; TT, thrombin time; UA, uric acid; WBC, white blood cell.

^a^Median (IQR); *n* (%); mean ± SD.

^b^Wilcoxon rank sum test.

^c^Pearson’s chi‐squared test.

^d^Welch Two sample *t*‐test.

^e^Fisher’s exact test.

Table 1 compares the baseline characteristics between the two groups. Demographic variables (age and gender) and medical history (hypertension, diabetes, smoking, etc.) showed no statistically significant differences between groups (all *p*  > 0.05).

In contrast, multiple laboratory indicators exhibited significant intergroup differences. Myocardial injury markers (NT‐proBNP, hs‐CTnI, and MYO) were substantially higher in the high PIV group. WBC, PLT, and several inflammation‐related markers (CRP, neutrophil, lymphocyte, monocyte, ALB, ALT, AST, and LDH) also differed significantly, with most of these markers showing higher levels in the high PIV group. Additionally, BUN and LDH were elevated in the high PIV group, suggesting potential renal impairment or systemic inflammatory changes.

Then, the Boruta algorithm was utilized for the variable selection of factors potentially influencing postoperative pulmonary artery pressure and perforation diameter, as presented in Tables S1 and S2. It was found that hs‐CTnI, Myo, WBC, PLT, neutrophils, lymphocytes, monocytes, CRP, ALB, ALT, AST, TBIL, PT, D‐D, APTT, BUN, and LDH were associated with postoperative pulmonary artery pressure and perforation diameter. These variables were subsequently ranked separately, and it was identified that TBIL, ALB, and ALT played an important role in postoperative pulmonary artery pressure, while ALB and ALT exerted a significant impact on perforation diameter, as shown in Figure 1. The statistical significance of these variables suggests that they are correlated with pulmonary hypertension and interventricular septal perforation area in patients who underwent transcatheter closure for postmyocardial infarction VSR.

Figure 1Importance of various features in classification models. (A, B) Ranking of clinical variables for predicting perioperative complications by Boruta algorithm. The plot demonstrates a boxplot of important attributes in color green, tentative attributes in yellow, unimportant attributes in red, and shadow attributes in blue, respectively. The vertical axis lists the name of each variable and the horizontal axis is the *Z*‐value. (C, D) A history graph of each decision to accept or reject by the random forest in the Boruta algorithm. The accepted attributes (green) have distinctly higher importance than the other attributes.

**Figure:**
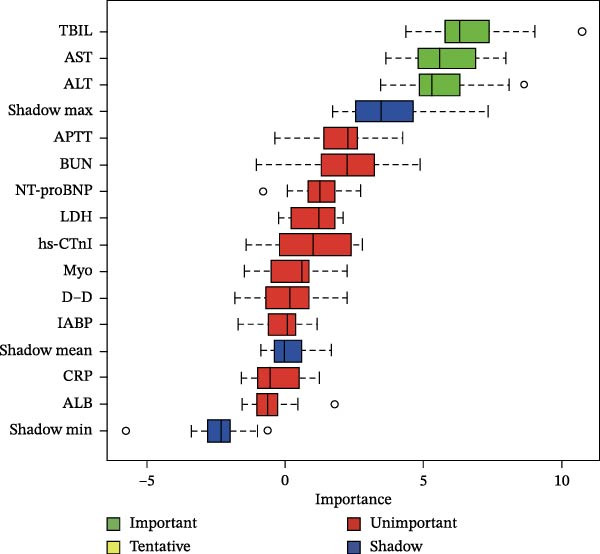
(A)

**Figure:**
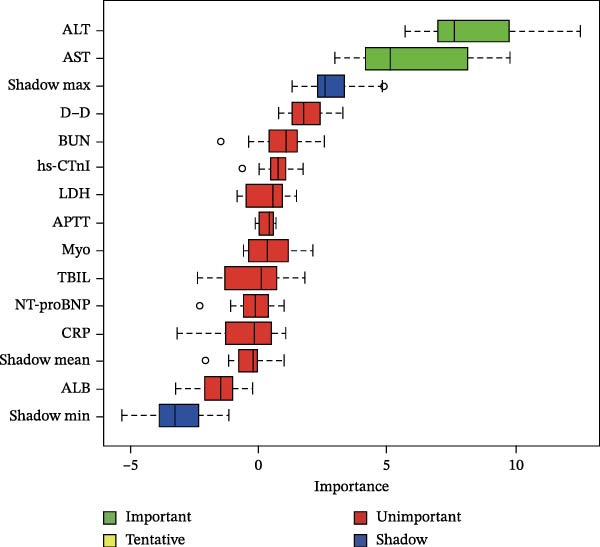
(B)

**Figure:**
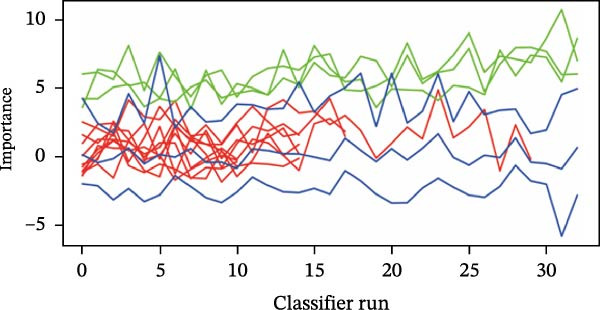
(C)

**Figure:**
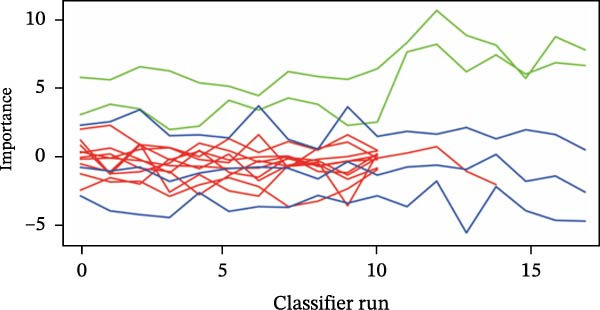
(D)

### 3.2. Incremental Predictive Value of PIV/100

To examine whether PIV/100 provides incremental discrimination beyond simpler inflammatory metrics derived from its components, we compared C‐index (AUC) values using an identical baseline covariate set (age, gender, BNP, EF, MI‐to‐operation time, and perforative parts) and then added one inflammatory metric at a time. In the full cohort (*N* = 133; events = 55), the baseline model achieved an AUC of 0.715 (95% CI: 0.625–0.804). Adding PIV/100 increased the AUC to 0.738 (95% CI: 0.651–0.826); however, the improvement did not reach statistical significance by DeLong’s test (*p* = 0.130). AUCs for models augmented with neutrophil count, NLR, PLT count, monocyte count, and lymphocyte count are reported in Table S3. Collectively, PIV/100 showed a modest numerical improvement in discrimination, warranting validation in larger cohorts.

### 3.3. Analysis of GAMs

After fitting the linear regression model, no significant association was found between PIV/100 and *Δ*PASP (*β* = −0.140, 95% CI: −0.316 to 0.035; *p* = 0.119). We, therefore, applied a GAM to explore potential nonlinearity between PIV/100 and *Δ*PASP, which suggested a possible nonlinear pattern (Figure 2A). Using segmented regression, an inflection point around PIV/100 approximately 6.36 was identified in the primary analysis. This value was then fixed as the break point for subsequent piecewise linear regression to quantify associations below and above the break point, and its stability was further examined in sensitivity analyses (Tables S4–S8). The threshold‐effect analysis of PIV/100 on *Δ*PASP is presented in Table 2.

Figure 2Relationship between PIV/100 and hemodynamic parameters. (A) Variation of *Δ*PASP (mm Hg) as a function of PIV/100, depicted by a blue curve with confidence interval bands shown as dotted lines. (B) Measurement of perforative diameter about PIV/100, also represented by a blue curve with confidence interval bands indicated as dotted lines.

**Figure:**
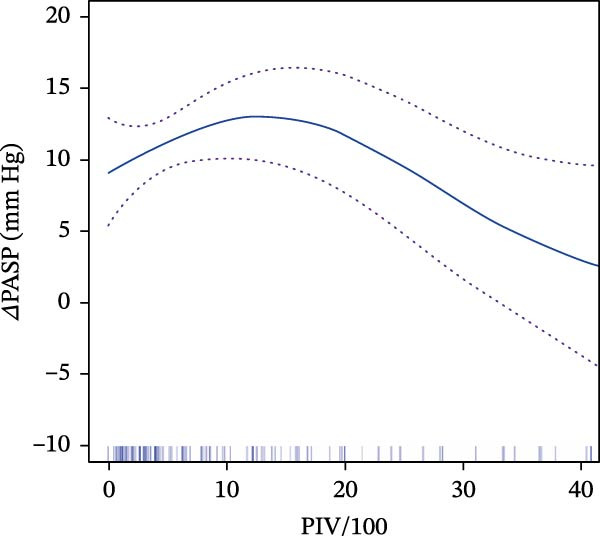
(A)

**Figure:**
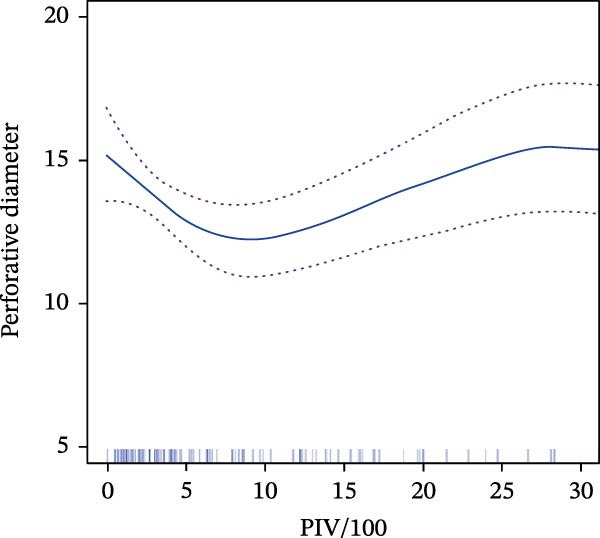
(B)

**Table 2:** Threshold effect analysis of PIV/100 on *Δ*PASP.

Row	Model 1		Model 2		Model 3	
	Beta (95% CI)	*p*‐Value	Beta (95% CI)	*p*‐Value	Beta (95% CI)	*p*‐Value
Fitting by a standard Linear regression model						
Total	−0.14 (−0.32, 0.04)	0.119	−0.11 (−0.29, 0.08)	0.253	−0.12 (−0.30, 0.05)	0.166
Fitted by a piecewise Linear regression model (break point = 6.36)						
PIV/100 <6.36	1.36 (0.05, 2.66)	0.041	1.15 (−0.19, 2.49)	0.091	1.25 (−0.07, 2.58)	0.063
PIV/100 ≥6.36	−0.30 (−0.52, −0.08)	0.008	−0.25 (−0.48, −0.01)	0.038	−0.27 (−0.49, −0.05)	0.017
Log likelihood ratio	—	0.023	—	0.058	—	0.035

*Note:* Model 1: nonadjusted; Model 2: adjusted for AST, ALT, and TBIL; Model 3: adjusted for age, gender, perforative parts, and perforative diameter.

Abbreviations: ALT, alanine aminotransferase; AST, aspartate aminotransferase; TBIL, total bilirubin.

Following identification of the inflection point (PIV/100 = 6.36), piecewise linear regression was used to quantify associations across intervals. In the lower interval (PIV/100 <6.36), a positive association was observed in the unadjusted model (*β* = 1.36, 95% CI: 0.05–2.66; *p* = 0.041). After adjustment for hepatic function markers (AST, ALT, and TBIL), the association was attenuated and became non‐significant (*β* = 1.15, 95% CI: −0.19 to 2.49; *p* = 0.091). Further adjustment for demographic and clinical characteristics (age, gender, perforation sites, and diameter) yielded a similar non‐significant result (*β* = 1.25, 95% CI: −0.07 to 2.58; *p* = 0.063), suggesting that the observed association may be influenced by confounding and should be interpreted cautiously.

In contrast, when PIV/100 is ≥6.36, the relationship with ΔPASP becomes negative, with each unit increase leading to a decrease in ΔPASP of roughly 0.30 mmHg (*β* = −0.30, 95% CI: −0.52, −0.08, *p* = 0.008). This relationship persisted with undiminished statistical significance following sequential adjustment for hepatic markers (*β* = −0.25, 95% CI: −0.48, −0.01, *p* = 0.038) and a comprehensive set of clinical covariates (*β* = −0.27, 95% CI: −0.49, −0.05, *p* = 0.017). Across progressively adjusted models, an elevated PIV/100 above the exploratory break point (~6.36) remained associated with a smaller *Δ*PASP after closure in the primary cohort, suggesting an attenuated pulmonary hemodynamic response.

Further analysis of the perforation diameter using a standard linear regression model showed no significant linear relationship with PIV/100 (*β* = 0.02, 95% CI: −0.03, 0.08, *p* = 0.441). Investigation using a GAM revealed a significant nonlinear, approximately inverted U‐shaped relationship (Figure 2B), prompting further analysis with a piecewise linear regression model at the identified threshold of PIV/100 = 6.36. The threshold‐effect analysis of PIV/100 on perforation diameter is presented in Table 3.

**Table 3:** Threshold effect analysis of PIV/100 on perforative diameter.

Row	Model 1		Model 2		Model 3	
	Beta (95% CI)	*p*‐Value	Beta (95% CI)	*p*‐Value	Beta (95% CI)	*p*‐Value
Fitting by a standard Linear regression model						
Total	0.02 (−0.03, 0.08)	0.441	−0.001 (−0.06, 0.06)	0.979	0.006 (−0.064, 0.075)	0.873
Fitting by a piecewise linear regression model (break point = 6.36)						
PIV/100 <6.36	−0.50 (−0.90, −0.11)	0.013	−0.47 (−0.87, −0.07)	0.021	−0.50 (−0.92, −0.07)	0.021
PIV/100 ≥6.36	0.08 (0.01, 0.14)	0.026	0.05 (−0.02, 0.12)	0.151	0.06 (−0.02, 0.14)	0.151
Log likelihood ratio	—	0.009	—	0.019	—	0.016

*Note:* Model 1: nonadjusted; Model 2: adjusted for AST and ALT; Model 3: adjusted for gender, age, BNP, hs‐CTnI, and Myo. BNP, prohormone of brain natriuretic peptide; CTnI, high‐sensitivity cardiac troponin I; Troponin I, cardiac troponin I.

Abbreviations: ALT, alanine aminotransferase; AST, aspartate aminotransferase; Myo, myoglobin.

The piecewise regression analysis delineated distinct associations across the PIV/100 spectrum. In the lower interval (PIV/100 <6.36), a significant negative association was observed in the unadjusted model (*β* = −0.50, 95% CI: −0.90, −0.11, *p* = 0.013). This association remained robust and statistically significant after sequential adjustment for hepatic enzymes (Model 2: *β* = −0.47, 95% CI: −0.87, −0.07, *p* = 0.021) and a broader set of clinical covariates (Model 3: *β* = −0.50, 95% CI: −0.92, −0.07, *p* = 0.021), indicating a stable and independent inverse relationship between PIV/100 and perforation diameter at lower inflammatory levels.

Conversely, in the upper interval (PIV/100 ≥6.36), the initial unadjusted model suggested a positive association (*β* = 0.08, 95% CI: 0.01–0.14, *p* = 0.026). However, this association was attenuated and lost statistical significance after adjusting for AST and ALT (Model 2: *β* = 0.05, 95% CI: −0.02, 0.12, *p* = 0.151) and further clinical variables (Model 3: *β* = 0.06, 95% CI: −0.02, 0.14, *p* = 0.151). The consistent non‐significance following adjustment indicates that no significant independent positive association exists between PIV/100 and perforation diameter in the high inflammatory range.

In summary, PIV/100 exhibits a clear threshold effect on perforation diameter. A stable, independent negative association is present at lower PIV/100 levels (<6.36), whereas no significant independent positive association persists at higher levels (≥6.36) after accounting for key clinical confounders.

### 3.4. Sensitivity Analyses for Systemic Inflammation (CRP–Based)

To mitigate potential confounding by systemic inflammatory burden, we performed two complementary sensitivity strategies. First, we excluded patients in the highest CRP quartile (CRP Q4; P75 = 57.47), retaining 99 patients, and reran the main modeling pipeline and aligned threshold analyses (Tables S4–S6). In this restricted cohort, the piecewise model did not provide a significantly better fit than the linear model in fully adjusted analyses (Model 3: LLR *p* = 0.296 for *Δ*PASP and *p* = 0.148 for perforative diameter). Second, in the full cohort we additionally adjusted for log(CRP + 1) (Model 4; Tables S7–S8). After this adjustment, the piecewise vs. linear fit advantage remained limited (Model 4: LLR *p* = 0.182 for *Δ*PASP and *p* = 0.056 for perforative diameter). These results support interpreting the break point as exploratory and potentially influenced by systemic inflammatory burden.

## 4. Discussion

VSR, a devastating mechanical complication following AMI, maintains a high mortality rate even in the reperfusion era. Although transcatheter closure offers a minimally invasive treatment option [18], the heterogeneity of patient outcomes remains a significant clinical challenge. Therefore, identifying biomarkers capable of accurately predicting postoperative pathophysiological changes is crucial. This study represents the first systematic investigation of the association between the systemic inflammatory marker—PIV—and perforation diameter in VSR patients, as well as postoperative changes in PASP (*Δ*PASP). Employing an advanced statistical strategy combining GAMs with piecewise linear regression, we observed a potential nonlinear pattern between PIV/100 and these outcomes, with an exploratory inflection point around 6.36. This finding provides a novel perspective on understanding the complex role of inflammation in the evolution of hemodynamics and anatomical structure following VSR.

A key observation was that the association between PIV/100 and *Δ*PASP did not appear strictly linear. When PIV/100 was below 6.36, the positive association with *Δ*PASP became nonsignificant after adjustment for hepatic enzymes, suggesting that the relationship may be sensitive to confounding by overall clinical status. In contrast, when PIV/100 exceeded 6.36, an inverse association with *Δ*PASP was observed and remained statistically significant after multivariable adjustment in the primary cohort (*β* = −0.27, *p* = 0.017). However, this break point effect should be interpreted cautiously given that it was attenuated in analyses accounting for systemic inflammatory burden. This finding carries significant pathophysiological implications: it suggests that while a moderate inflammatory response may participate in beneficial repair processes, a systemic inflammatory level surpassing a specific threshold pivots towards detrimental pathways. We speculate that a heightened immune milieu reflected by a high PIV may contribute to attenuated pulmonary hemodynamic improvement through several interconnected mechanisms. First, activated neutrophils may release neutrophil extracellular traps (NETs), which not only contribute to microvascular occlusion and amplify sterile inflammation but also directly damage endothelial cells, thereby exacerbating pulmonary endothelial dysfunction [19]. Second, high PLT counts, coupled with activated monocytes and neutrophils, foster the formation of PLT–leukocyte aggregates. These aggregates can physically obstruct the pulmonary microvasculature and serve as a potent source of pro‐inflammatory and pro‐fibrotic cytokines (e.g., TGF‐β and IL‐1β), fueling a vicious cycle of vascular inflammation and remodeling. Collectively, these processes may contribute to the attenuated regression of pulmonary hypertension observed in patients with higher PIV after closure.

Analysis of perforation diameter revealed a distinct threshold effect. At lower PIV/100 levels (<6.36), a significant negative correlation was observed, suggesting a moderate inflammatory response may help contain rupture expansion by facilitating reparative processes [20–22]. However, once PIV/100 exceeded this threshold, no independent association with diameter persisted after multivariable adjustment. This indicates that in high‐inflammatory states, the final defect size is predominantly determined by other potent factors, such as infarct extent and local mechanical forces [21, 23], rather than by the systemic inflammatory level per se.

Another observation was that Boruta screening identified AST and ALT as important variables related to the outcomes. This aligns strongly with research in cardiogenic shock [24], where elevated liver enzymes are sensitive markers of systemic hypoperfusion and end‐organ damage [25]. Therefore, elevated AST and ALT, consistent with the more severe clinical status in the high PIV group, serve as objective biomarkers of the systemic hypoperfusion and end‐organ damage characteristic of cardiogenic shock [26].

Our findings also underscore the clinical relevance of an elevated PIV, as it was associated with a more severe illness phenotype. Patients in the high PIV group required significantly more frequent use of IABP support (60.3% vs. 36.9%, *p* = 0.007) and showed a trend towards higher utilization of CRRT. This strongly suggests that a heightened systemic inflammatory state, as reflected by PIV, is correlated with greater hemodynamic instability and end‐organ dysfunction, necessitating more advanced mechanical circulatory and renal support. This association further validates PIV as a marker encompassing not only inflammation but also overall clinical severity in patients with VSR.

This study positions PIV as an inflammatory marker with potential clinical utility. The revelation of its nonlinear relationship implies that future clinical interpretation should avoid simplistic linear thinking of “lower is better” or “higher is worse.” For instance, it is plausible that patients with a PIV/100 >6.36 may benefit from closer monitoring for attenuated postoperative pulmonary pressure improvement. These findings suggest that patients with higher PIV/100 may warrant closer hemodynamic surveillance after closure in future studies. The prognostic value of PIV has been demonstrated in other cardiovascular conditions, such as STEMI, supporting its broader utility. However, it must be emphasized that PIV is currently in an exploratory stage, and its ability to independently guide clinical decisions awaits validation in prospective studies. Future efforts should focus on establishing multicenter, prospective cohorts and integrating PIV into comprehensive predictive models that include imaging, hemodynamic, and molecular biomarkers.

## 5. Limitations

This study has several limitations. First, its single‐center and retrospective design is inherently susceptible to selection bias. Second, the lack of quantitative data on myocardial infarct size is a significant limitation, as this factor is a fundamental driver of both VSR severity and the magnitude of the inflammatory response; its absence may introduce unquantifiable confounding. Specifically, we cannot rule out the possibility that the observed associations with PIV are partly mediated by unmeasured differences in infarct size between patients. Furthermore, the observational design precludes the establishment of causality.

## 6. Future Perspectives

Future research should simultaneously measure PIV and quantitatively assess infarct size (e.g., via cardiac magnetic resonance) to disentangle their independent contributions and potential mediating relationships. Additionally, multicenter and prospective studies are warranted to validate the prognostic utility of PIV and to integrate it with other imaging, hemodynamic, and molecular biomarkers into comprehensive predictive models.

## 7. Conclusion

This study reveals a significant nonlinear threshold relationship between PIV/100 and both postoperative pulmonary artery pressure changes and perforation diameter in VSR patients. Our findings illuminate the complex, context‐dependent role of systemic inflammation in the pathophysiology following VSR and emphasize the necessity of considering threshold effects in biomarker research. As an exploratory investigation, our results suggest PIV’s potential utility as an adjunct biomarker for identifying patient subgroups at elevated risk of complications. However, these observations require validation in larger and prospective cohorts to establish definitive clinical utility and reliable intervention thresholds.

## Author Contributions

**Xinlong Di and Zebin Lin**: writing – original draft, methodology, investigation, formal analysis, data curation. **Qingwang Hou and Tongfeng Chen**: writing – review and editing, methodology, investigation, formal analysis, data curation, conceptualization. **Xiaohu Wang and Chong Chen**: writing – review and editing, formal analysis, resources. **Yipin Zhao**, **Jianmin Tang**, and **Yuhao Liu**: writing – review and editing, methodology, formal analysis, data curation.

## Funding

This research was supported by Henan Provincial Key Technologies R&D Program (Grant 25202310242) and the Henan Provincial Medical Science and Technology Tackling Program (Grant LHGJ20240150).

## Ethics Statement

The study involving human participants has received approval from the Central China Fuwai Hospital of Zhengzhou University. It was carried out in compliance with local regulations and institutional guidelines. The Ethics Committee/Institutional Review Board waived the need for written informed consent from participants or their legal guardians/relatives due to the challenges of obtaining written consent in retrospective studies.

## Conflicts of Interest

The authors declare no conflicts of interest.

## Supporting Information

Additional supporting information can be found online in the Supporting Information section.

## Supporting information


**Supporting Information** Supporting Information is available for this study and provides additional methodological details and sensitivity analyses supporting the main findings. Tables S1 and S2 present the Boruta‐based variable selection results for *Δ*PASP and perforative diameter, respectively. Table S3 presents the comparison of C‐index and AUC for models incorporating PIV/100 and other inflammatory markers using DeLong tests. Table S4 presents the results of the rerun main analysis after exclusion of the highest CRP quartile. Tables S5 and S6 present the threshold‐effect analyses of PIV/100 on *Δ*PASP and perforative diameter, respectively, after exclusion of the highest CRP quartile, corresponding to Tables 2 and 3 in the main manuscript. Tables S7 and S8 further present full‐cohort sensitivity analyses of the threshold effects of PIV/100 on *Δ*PASP and perforative diameter with additional adjustment for log (CRP+1). Table S9 presents the covariate‐selection sensitivity analysis comparing the prespecified fully adjusted model with the Boruta‐augmented model.

## Data Availability

The datasets generated and/or analyzed during the current study are available from the corresponding author upon reasonable request.
