# Supplementary material for: Pan‐Immune‐Inflammation Value: Related to Perforation Diameter and Pulmonary Artery Pressure in Ventricular Septal Rupture Patients
Source: Mediators Inflamm. 2026 Apr 8;2026:5407966. doi: 10.1155/mi/5407966 (PMC13058817; doi:10.1155/mi/5407966)
Supplement: Supplementary file 1 — Supporting Information Supporting Information is available for this study and provides additional methodological details and sensitivity analyses supporting the main findings. Tables S1 and S2 present the Boruta‐based variable selection results for ΔPASP and perforative diameter, respectively. Table S3 presents the comparison of C‐index and AUC for models incorporating PIV/100 and other inflammatory markers using DeLong tests. Table S4 presents the results of the rerun main analysis after exclusion of the highest CRP quartile. Tables S5 and S6 present the threshold‐effect analyses of PIV/100 on ΔPASP and perforative diameter, respectively, after exclusion of the highest CRP quartile, corresponding to Tables 2 and 3 in the main manuscript. Tables S7 and S8 further present full‐cohort sensitivity analyses of the threshold effects of PIV/100 on ΔPASP and perforative diameter with additional adjustment for log (CRP+1). Table S9 presents the covariate‐selection sensitivity analysis comparing the prespecified fully adjusted model with the Boruta‐augmented model. [file MI-2026-5407966-s001.zip › Table_S4_CRPQ4_excluded_ReRun_MainPipeline.docx]

**Supplementary Tables: Sensitivity analysis excluding the highest CRP quartile (CRP Q4)**

**Table S4A. Summary of CRP Q4 exclusion (CRP ≥ P75).**

| **Sensitivity set** | **CRP P75 cutoff** | **Total N** | **Excluded N** | **Remaining N** |
| --- | --- | --- | --- | --- |
| Exclude CRP Q4 (CRP < P75) | 57.47 | 133 | 34 | 99 |

**Table S4B. Re-running the manuscript’s main modeling pipeline after CRP Q4 exclusion (Linear → GAM → segmented → LRT).**

| **Outcome** | **N (after CRP Q4 excl.)** | **Linear regression (piv_100)** | **GAM nonlinearity** | **Segmented breakpoint (estimated)** | **LRT segmented vs linear** | **Fixed breakpoint 6.36 (hinge)** |
| --- | --- | --- | --- | --- | --- | --- |
| Perforative diameter | 99 | β=0.0285; SE=0.0391; p=0.4680 | edf=0.0003; p=0.5596 | 32.568 (SE=11.654) | p=0.5177 | x1 β=-0.2394 (p=0.3149); x2 β=0.0675 (p=0.1958) |
| ΔPASP (preoperative − postoperative) | 99 | β=-0.1605; SE=0.1345; p=0.2358 | edf=0.0005; p=0.4491 | 22.856 (SE=8.197) | p=0.1384 | x1 β=0.6846 (p=0.4214); x2 β=-0.2832 (p=0.1216) |

Abbreviations: PIV, pan-immune-inflammation value; PIV/100 corresponds to piv_100. CRP Q4 indicates the highest quartile of CRP (cutoff = P75). ΔPASP = preoperative PASP − postoperative PASP (improvement as positive). GAM, generalized additive model; LRT, likelihood-ratio test. Hinge model uses a fixed breakpoint at PIV/100 = 6.36 with x1=min(PIV/100,6.36) and x2=max(PIV/100−6.36,0).
