# Supplementary material for: Pan‐Immune‐Inflammation Value: Related to Perforation Diameter and Pulmonary Artery Pressure in Ventricular Septal Rupture Patients
Source: Mediators Inflamm. 2026 Apr 8;2026:5407966. doi: 10.1155/mi/5407966 (PMC13058817; doi:10.1155/mi/5407966)
Supplement: Supplementary file 1 — Supporting Information Supporting Information is available for this study and provides additional methodological details and sensitivity analyses supporting the main findings. Tables S1 and S2 present the Boruta‐based variable selection results for ΔPASP and perforative diameter, respectively. Table S3 presents the comparison of C‐index and AUC for models incorporating PIV/100 and other inflammatory markers using DeLong tests. Table S4 presents the results of the rerun main analysis after exclusion of the highest CRP quartile. Tables S5 and S6 present the threshold‐effect analyses of PIV/100 on ΔPASP and perforative diameter, respectively, after exclusion of the highest CRP quartile, corresponding to Tables 2 and 3 in the main manuscript. Tables S7 and S8 further present full‐cohort sensitivity analyses of the threshold effects of PIV/100 on ΔPASP and perforative diameter with additional adjustment for log (CRP+1). Table S9 presents the covariate‐selection sensitivity analysis comparing the prespecified fully adjusted model with the Boruta‐augmented model. [file MI-2026-5407966-s001.zip › Table_S7_FullCohort_Model4_logCRP_Aligned_Table2_DeltaPASP.docx]

**Table S7. Sensitivity analysis (full cohort): Threshold effect of PIV/100 on ΔPASP with additional adjustment for log(CRP+1) (Model 4).**

| **Row** | **Model 1 Beta (95% CI)** | **Model 1 P-value** | **Model 2 Beta (95% CI)** | **Model 2 P-value** | **Model 3 Beta (95% CI)** | **Model 3 P-value** | **Model 4 (+logCRP) Beta (95% CI)** | **Model 4 P-value** |
| --- | --- | --- | --- | --- | --- | --- | --- | --- |
| Fitting by standard linear regression model |  |  |  |  |  |  |  |  |
| Total | -0.104 (-0.283, 0.075) | 0.256 | -0.052 (-0.241, 0.137) | 0.59 | -0.108 (-0.286, 0.069) | 0.234 | -0.144 (-0.335, 0.048) | 0.143 |
| Fitting by piecewise linear regression model (breakpoint = 6.36) |  |  |  |  |  |  |  |  |
| PIV/100 < 6.36 | 1.089 (-0.302, 2.480) | 0.127 | 1.152 (-0.272, 2.576) | 0.115 | 0.944 (-0.437, 2.324) | 0.183 | 0.808 (-0.641, 2.258) | 0.277 |
| PIV/100 ≥ 6.36 | 0.867 (-0.426, 2.160) | 0.191 | 0.980 (-0.346, 2.305) | 0.15 | 0.730 (-0.552, 2.012) | 0.266 | 0.582 (-0.785, 1.950) | 0.405 |
| Log likelihood ratio | 0.088 |  | 0.089 |  | 0.123 |  | 0.182 |  |

Notes: Breakpoint fixed at PIV/100 = 6.36. Model 4 = Model 3 + log(CRP+1). Log likelihood ratio values compare piecewise vs standard linear models within the same adjustment set.
